# Supplementary material for: Development of Hemispherical 3D Models of Human Brain and B Cell Lymphomas Using On-Chip Cell Dome System
Source: Bioengineering (Basel). 2024 Dec 23;11(12):1303. doi: 10.3390/bioengineering11121303 (PMC11727638; doi:10.3390/bioengineering11121303)
Supplement: Supplementary file 1 [file bioengineering-11-01303-s001.zip › bioengineering-3371585-supplementary.pdf]

Supplementary information

Development of Hemispherical 3D Models of Human Brain and B Cell Lymphomas Using On-Chip Cell Dome System

Ryotaro Kazama, Rina Ishikawa and Shinji Sakai *

Graduate School of Engineering Science, Osaka University, 1-3 Machikaneyama, Toyonaka, Osaka 560-8531, Japan.

* Correspondence: sakai.shinji.es@osaka-u.ac.jp; Tel: +81-6-6850-6252

Table S1. The mean fluorescence intensities of TK or KML-1 cells, cultured in a 2D flask without immunostaining, 2D cultured cells immunostained with anti-CD19, and cells cultured in Cell Domes or high-density Cell Domes immunostained with anti-CD19 (n = 3).

Sample	Mean intensity of surface CD19 Expression on TK cells [a.u.]	Mean intensity of surface CD19 expression on KML-1 cells [a.u.]
2D flask without anti-CD19	$0.9 \pm 0.4 \times 10^4$	$0.4 \pm 0.1 \times 10^3$
2D flask with anti-CD19	$3.4 \pm 1.0 \times 10^4$	$2.5 \pm 1.0 \times 10^3$
For 2 days in Cell Domes with anti-CD19	$3.2 \pm 0.4 \times 10^4$	$3.1 \pm 0.4 \times 10^3$
For 7 days in Cell Domes with anti-CD19	$2.0 \pm 0.5 \times 10^4$	$3.0 \pm 0.3 \times 10^3$
For 10 days in Cell Domes with anti-CD19	$2.1 \pm 0.5 \times 10^4$	$2.2 \pm 0.4 \times 10^3$
For 3 days in high-density Cell Domes with anti-CD19	$4.6 \pm 0.2 \times 10^4$	$3.8 \pm 0.4 \times 10^3$

Table S2. The mean fluorescence intensities of TK or KML-1 cells, cultured in a 2D flask without immunostaining, 2D cultured cells immunostained with anti-CD20, and cells cultured in Cell Domes or high-density Cell Domes immunostained with anti-CD20 (n = 3).

Sample	Mean intensity of surface CD20 expression on TK cells [a.u.]	Mean intensity of surface CD20 expression on KML-1 cells [a.u.]
2D flask without anti-CD20	$5.0 \pm 2.0 \times 10^4$	$0.4 \pm 0.1 \times 10^4$
2D flask with anti-CD20	$19.4 \pm 1.2 \times 10^4$	$18.5 \pm 6.2 \times 10^4$
For 2 days in Cell Domes with anti-CD20	$46.3 \pm 17.4 \times 10^4$	$15.7 \pm 0.3 \times 10^4$
For 7 days in Cell Domes with anti-CD20	$28.5 \pm 5.2 \times 10^4$	$8.9 \pm 0.4 \times 10^4$
For 10 days in Cell Domes with anti-CD20	$35.2 \pm 6.7 \times 10^4$	$5.0 \pm 1.3 \times 10^4$
For 3 days in high-density Cell Domes with anti-CD20	$93.8 \pm 4.1 \times 10^4$	$12.1 \pm 1.7 \times 10^4$
